# Pneumocephalus Associated with Cerebrospinal Fluid Fistula as a Complication of Spinal Surgery: A Case Report

**DOI:** 10.1155/2010/328103

**Published:** 2010-06-14

**Authors:** Ken Sasaki, Tomoyuki Matsumoto, Toshiyuki Mizuno, Shinichi Ikuta, Toshihiro Akisue, Hiroyuki Fujioka, Minoru Doita, Masahiro Kurosaka, Ryosuke Kuroda

**Affiliations:** ^1^Department of Orthopaedic Surgery, Graduate School of Medicine, Kobe University, 7-5-2 Kusunoki-cho, Chou-ku, Kobe 650-0017, Japan; ^2^Department of Orthopaedic Surgery, Rokko Hospital, Kobe 657-0022, Japan

## Abstract

Pneumocephalus is a well-known condition following head trauma, but is rare as an injury or as a result of surgery of the spine. We present a 76-year-old patient with a rare case of pneumocephalus associated with a cerebrospinal fluid fistula as a complication of surgical treatment for cervical myelopathy. Although cerebrospinal fluid leakage was noted and the injured dura was carefully sutured at operation, tension pneumocephalus occurred. The resultant pneumocephalus was diagnosed based on neurogenic symptoms including sudden convulsion, head radiograph, and computed tomography scan. The benign course of the pneumocephalus postdiagnosis did not require secondary operation.

## 1. Introduction

Pneumocephalus is a rare phenomenon indicating a pathological presence of intracranial air. Although a great number of cases has been reported following head trauma [1], our case occurring following spinal surgery is rare. We present a patient with pneumocephalus associated with a cerebrospinal fluid fistula as a complication of surgical treatment for cervical myelopathy.

## 2. Case Report

A 76-year-old man presented with numbness in the upper and lower limbs in addition to neck pain of a two months' duration. He was able to support himself and had no gait disturbance. Joint position was present in all four limbs. More recently, he had experienced frequent urination and urinary retention. 

Physical examination revealed a sensory disturbance of a glove and stocking type, slight weakness of the musculature in the upper and lower extremities (grade 4-5 bilaterally, as determined by manual muscle testing), and exaggerated deep tendon reflexes in the lower extremities. Cranial nerve function was intact. The patient showed no clinical, radiologic, or serologic evidence of rheumatoid arthritis according to criteria of the American Rheumatism Association. 

**Figure 1:**
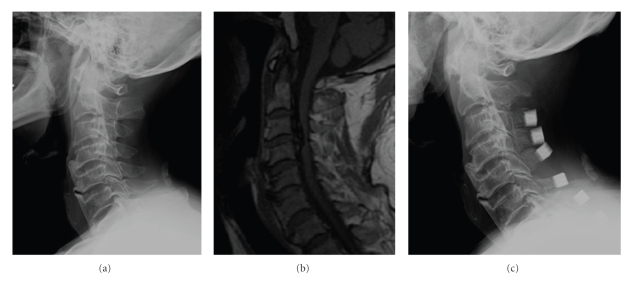
Preoperative image ((a): plain radiograph, lateral image, (b): MRI, sagittal image, and T1 weighted image). (a) Preoperative plain radiograph demonstrates significant multilevel cervical spondylosis. (b) In preoperative MRI, spinal cord compression at C3-4 due to OPLL is found. (c) Laminoplasty is performed using hydroxyapatite spacers from C3 to C7. MRI: Magnetic resonance imaging. OPLL: Ossification of posterior longitudinal ligament.

Plain radiographs (Figure 1(a)) revealed progression of subaxial degeneration disease, progressive cervical fusion with C4-6 and C3-4 mixed type of ossification of the posterior longitudinal ligament (OPLL). Magnetic resonance imaging (MRI) of the cervical spine (Figure 1(b)) confirmed the presence of OPLL with low signal intensity on T1 and T2-weighted images and the severe compression to the spinal cord. Myelogram showed incomplete block at C3-4 level of the omnipaque while postmyelography computed tomography of C3/4 showed severe spinal cord compression due to OPLL. The patient was diagnosed with cervical myelopathy due to significant multilevel cervical spondylosis and OPLL. He subsequently underwent Kurokawa's procedure [2] using hydroxyapatite spacers performed with T-saw thread wire from C3 to C7 (Figure 1(c)). In the operation, CSF leakage was noted and sutured carefully with a fat patch. No CSF leakage was observed after repair. One drain without suction was placed subcutaneously.

**Figure 2:**
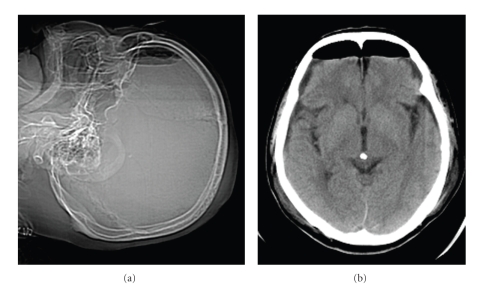
Head image ((a): Plain radiograph, (b): CT). (a) Plain radiograph demonstrates significant intracranial air. (b) CT reveals massive pneumocephalus involving the subarachnoid spaces. CT: Computed tomography.

**Figure 3:**
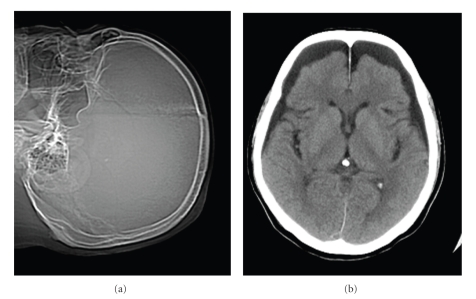
One month postoperative head image ((a): Plain radiograph, (b): CT). (a) Plain radiograph demonstrates the disappearance of intracranial air. (b) CT one month after surgery demonstrates the disappearance of air in the overall space. (c) CT: Computed tomography.

In the immediate postoperative period, the patient's recovery was uneventful. The drain was removed the next day and routine antibiotics (Cefazolin 2 g/days) were administered for 3 days. Although vital signs and laboratory data were within normal limits, two days after surgery the patient developed severe headache, nausea, and sudden convulsion. Plain radiograph and computed tomography (CT) of the head revealed massive pneumocephalus involving the subarachnoid spaces without any midline shift (Figures 2(a) and 2(b)). We aspirated the serous bloody fluid under the wound, which was negative in culture. CSF fistula was stopped until 4 days after surgery. Complete *bed rest* for one week resulted in complete resolution of his symptoms. Conservative treatment led to no further convulsion and no other symptoms. Plain radiograph and CT one month after surgery (Figures 3(a) and 3(b)) demonstrated the disappearance of air in the overall space. No epilepsy or abnormal brain waves were noted up to one month after the onset of the initial convulsions, suggesting those convulsions were due not to conventional epilepsy but a massive pneumocephalus. Six months after surgery, sensory disturbance had almost disappeared and weakness and spasticity in the extremities were improved. The Japanese Orthopaedic Association scores for cervical myelopathy (full score, 17 points) [3] had improved from 12 to 14 points. 

## 3. Discussion

Pneumocephalus was first described in 1866 [4]. Air or gas can be localized in the subdural, subarachnoid, epidural, intraventricular, or intraparenchymal spaces. Pneumocephalus can be classified by etiology into two broad categories of traumatic and nontraumatic. The most frequent cause of pneumocephalus is head trauma [1], accounting for 74% of all cases, followed by intracranial neoplasms, infections, neurosurgery, paranasal sinus surgery, and diagnostic or neurosurgical interventions such as pneumocephalography or lumbar puncture [5]. To our knowledge, only a few cases of pneumocephalus as a complication of spinal surgery have been reported [6, 7]. In addition, severe symptoms of the cranial nerve such as convulsion due to a massive pneumocephalus occurring after spinal surgery in which an injured dura was repaired have never been reported.

CT scan and X-rays are useful in the detection of pneumocephalus. However, the exact incidence of this complication is unknown because cranial imaging after spinal surgery is not routinely performed. In our case, CT scan and X-ray were performed two days after surgery due to the sudden convulsions. This paper suggests that cranial images after spinal surgery should be taken if dural injury or CSF leakage occurs during the operation. 

Many causes of postoperative tension pneumocephalus have been reported. The developmental mechanism of pneumocephalus is mainly based on two factors: a reduction in intracranial pressure and the presence of a defect in the dura [8]. It is caused by either a ball-valve mechanism that allows air to enter but not to exit, or by CSF leakage which creates a negative pressure with subsequent air entry [9, 10]. In our case, intracranial air might have increased gradually through the dural tear due to CSF leakage. Therefore, the disappearance of the CSF fistula as a result of conservative treatment (bed rest) resulted in complete resolution.

When pneumocephalus occurs, the course is usually resolved in the majority of cases within 2 to 3 weeks by conservative treatment [11]. If CSF fistula persists after one week of conservative management, active intervention is required. In our patient, the CSF fistula was stopped until 4 days after surgery and conservative treatment, including bed rest and avoiding manipulations that increase intracranial pressure, led to no further convulsions and no other symptoms. The intracranial air dispersed spontaneously within two weeks of surgery. Our case suggests that pneumocephalus can occur in spite of dural tear repair at the time of surgery. Spine surgeons should recognize pneumocephalus as a complication of spine surgery even if the injured dura is carefully repaired.

## 4. Conclusion

We presented a patient with a massive pneumocephalus after cervical surgery because of a dural tear despite its careful repair during operation. To prevent such a severe complication, it is essential that surgeons be aware of this possible complication after spine surgery, even if the dural tear was carefully repaired.
